# Predictors and clinical outcomes of true mitral stenosis in patients undergoing transcatheter aortic valve implantation

**DOI:** 10.1093/ehjimp/qyae109

**Published:** 2024-10-23

**Authors:** Mitsuki Yamaga, Masaki Izumo, Yukio Sato, Tatsuro Shoji, Daisuke Miyahara, Yoshikuni Kobayashi, Takahiko Kai, Taishi Okuno, Shingo Kuwata, Masashi Koga, Yasuhiro Tanabe, Yoshihiro J Akashi

**Affiliations:** Department of Cardiology, St. Marianna University School of Medicine, 2-16-1, Sugao, Miyamae-ku, Kawasaki 216-8511, Japan; Department of Cardiology, Mishuku Hospital, Tokyo, Japan; Department of Cardiology, St. Marianna University School of Medicine, 2-16-1, Sugao, Miyamae-ku, Kawasaki 216-8511, Japan; Department of Cardiology, St. Marianna University School of Medicine, 2-16-1, Sugao, Miyamae-ku, Kawasaki 216-8511, Japan; Department of Cardiology, St. Marianna University School of Medicine, 2-16-1, Sugao, Miyamae-ku, Kawasaki 216-8511, Japan; Department of Cardiology, St. Marianna University School of Medicine, 2-16-1, Sugao, Miyamae-ku, Kawasaki 216-8511, Japan; Department of Cardiology, St. Marianna University School of Medicine, 2-16-1, Sugao, Miyamae-ku, Kawasaki 216-8511, Japan; Department of Cardiology, St. Marianna University School of Medicine, 2-16-1, Sugao, Miyamae-ku, Kawasaki 216-8511, Japan; Department of Cardiology, St. Marianna University School of Medicine, 2-16-1, Sugao, Miyamae-ku, Kawasaki 216-8511, Japan; Department of Cardiology, St. Marianna University School of Medicine, 2-16-1, Sugao, Miyamae-ku, Kawasaki 216-8511, Japan; Department of Cardiology, St. Marianna University School of Medicine, 2-16-1, Sugao, Miyamae-ku, Kawasaki 216-8511, Japan; Department of Cardiology, St. Marianna University School of Medicine, 2-16-1, Sugao, Miyamae-ku, Kawasaki 216-8511, Japan; Department of Cardiology, St. Marianna University School of Medicine, 2-16-1, Sugao, Miyamae-ku, Kawasaki 216-8511, Japan

**Keywords:** aortic stenosis, mitral stenosis, transcatheter aortic valve implantation

## Abstract

**Aims:**

Predictors of true degenerative mitral stenosis (MS) in patients with aortic stenosis who underwent transcatheter aortic valve implantation (TAVI) remain unknown. This study aimed to investigate the predictors and prognostic value of true degenerative MS in this population.

**Methods and results:**

We retrospectively reviewed the records of 760 consecutive patients who underwent TAVI. The mitral valve area (MVA) was assessed using transthoracic echocardiography, and mitral valve calcification was assessed using multi-detector computed tomography. MS was defined as an MVA of ≤2.0 cm², and true MS was defined as moderate or severe MS following TAVI. In our TAVI cohort, we identified 72 (9.5%) patients with degenerative MS. Among these, true MS was observed in 38 (52.7%) patients. Echocardiographic data showed that the true MS group had a significantly lower MVA and higher trans-mitral gradient. The severity of mitral annular calcification was not significantly different between the two groups; however, the true MS group had significantly more posterior mitral leaflet and anterior mitral leaflet (AML) calcification. Multivariable logistic regression analysis showed that AML calcification was the independent predictor of true MS [adjusted odds ratio, 9.23; 95% confidence interval (CI) 2.84–29.9]. True MS was independently associated with poor prognosis (adjusted hazard ratio, 2.76; 95% CI 1.09–6.98).

**Conclusion:**

Approximately half of the patients with concomitant degenerative MS who underwent TAVI had true MS, which was associated with a poor prognosis. Computed tomographic analysis of AML calcification was useful for predicting true MS.

## Graphical Abstract

**Graphical Abstract:**
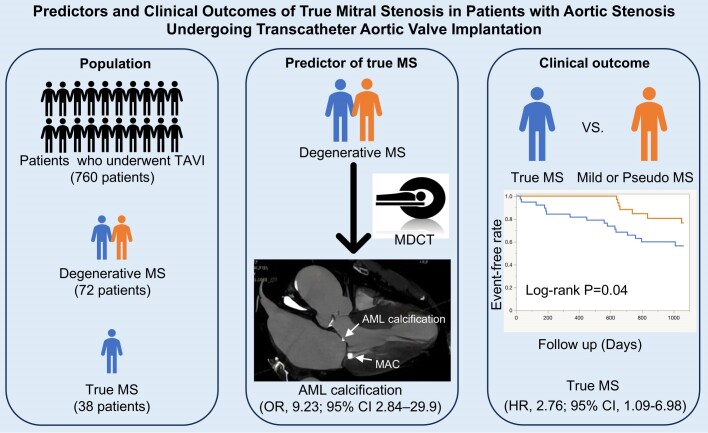

## Introduction

The number of patients with degenerative mitral stenosis (MS) increases with age. Mitral annular calcification (MAC), the main cause of degenerative MS, is more prevalent in older adults, patients with chronic kidney disease, and individuals with multiple cardiovascular risk factors, such as hypertension, diabetes mellitus, dyslipidaemia, and smoking.^1^ These factors are often shared by patients with aortic stenosis (AS). Almost half of the patients with severe AS reportedly have MAC. Notably, almost half of the patients with severe AS also exhibit MAC, leading to a frequent comorbidity of degenerative MS and AS.^2^ Many of these patients are at high risk for open-heart surgery due to advanced age, frailty, and multiple comorbidities.^3^ Transcatheter aortic valve implantation (TAVI) has emerged as a treatment option for high-risk patients with severe AS. Approximately 10% of patients undergoing TAVI have concomitant MS.^4^ However, the co-occurrence of MS and AS complicates the assessment of the severity of each condition due to their interactions, and treatment strategies for these patients remain unestablished.^5,6^ A recent study reported that some AS patients with concomitant MS [trans-mitral gradient (TMG) ≥ 4 mmHg] have increased mitral valve area (MVA) after aortic valve replacement (AVR) (surgical AVR or TAVI), overestimating MS severity. Conversely, there are patients with ‘true MS’ whose MVA does not increase after AVR, and these patients are associated with poor prognosis.^7^

Previous studies have reported a poor prognosis in patients with concomitant severe MS undergoing TAVI.^4^ In patients with combined AS and MS, careful treatment selection is required, considering the severity of MS and patient’s general condition before undergoing intervention for AS.^2,8^ However, the predictors of true MS are not well known, and risk stratification in this population is important for determining treatment strategies. Therefore, this study aimed to investigate the predictors of true MS and their effect on the clinical outcomes of patients with degenerative MS undergoing TAVI.

## Methods

### Study population

This retrospective observational study included 760 consecutive patients aged 18 years and older with severe AS who underwent TAVI at St. Marianna University Hospital, Kawasaki, Japan, between January 2016 and December 2021. The inclusion criteria were patients diagnosed with degenerative MS, characterized by calcium deposition extending from the annulus to the apex without fusion of the mitral commissure. The exclusion criteria were as follows: (i) rheumatic MS, (ii) congenital heart disease, (iii) hypertrophic cardiomyopathy, (iv) moderate or severe aortic regurgitation, (v) moderate or severe mitral regurgitation, (vi) left ventricular (LV) outflow tract obstruction, and (vii) a heart rate of >100 bpm. The last six conditions were excluded because they affected the accuracy of MVA measurements using the continuity equation. This study was approved by the Institutional Ethics Committee of St. Marianna University School of Medicine (no. 6336), and the need for informed consent was waived owing to the retrospective nature of the study.

### Transthoracic echocardiography and definitions of true MS

All patients underwent comprehensive two-dimensional and Doppler transthoracic echocardiography (TTE) according to the guidelines of the American Society of Echocardiography^9,10,11^ preoperatively, at discharge, at 1 year, and annually after TAVI. Because the TMG in this population must consider LV and left atrial compliance and may not reflect the exact severity of MS,^12^ this study defined an MVA of ≤2.0 cm² as MS. The MVA was measured using the continuity equation, and the severity of MS was defined as severe (≤1.0 cm²), moderate (1.0–1.5 cm²), or mild (1.5–2.0 cm²).^13^ In this study, a significant change was defined as a change of ≥0.1 cm² between pre-TAVI and post-TAVI discharge.^14^ True MS was defined as moderate or severe MS after TAVI.

### Assessment of mitral valve calcification

**Figure 1:**
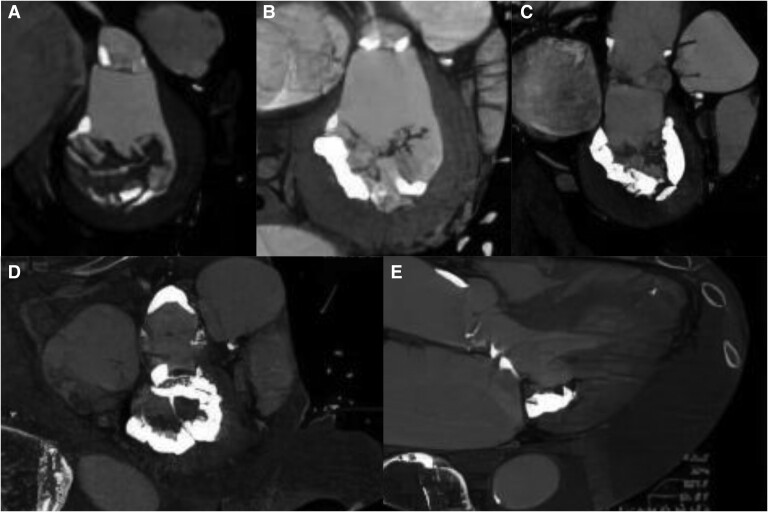
Qualitative evaluation of mitral valve calcification assessed using multi-detector CT. MAC severity was determined according to the visual circumferential involvement of the mitral annulus as follows: (*A*) mild (less than one-third of the annulus), (*B*) moderate (between one-third and one-half of the annulus), and (*C*) severe (more than half of the annulus). Valve leaflet calcification was identified by observing the mitral valve in two directions, the short axis (*D*) and long axis (*E*), to distinguish it from LV outflow tract calcification or MAC.

MAC was evaluated morphologically using multi-detector computed tomography (CT) (Aquilion One; Canon, Tokyo, Japan) before TAVI. CT data were processed for analysis using Ziostation2 software (Ziosoft, Tokyo, Japan). Three-dimensional analysis of this program was used to reconstruct the mitral valve in short-axis, long-axis, and commissural views, and the distribution of calcification was evaluated from multiple cross-sections. The severity of MAC was evaluated using the short-axis view and defined as the percentage of the area of calcification relative to the circumference of the mitral annulus: mild (<1/3), moderate (1/3–1/2), and severe (>1/2),^15^ as shown in *Figure 1A–C*. In addition, with reference to the cardiac CT-based assessment method for mitral valve calcification previously reported by Guerrero *et al.*,^16^ the commissures of the mitral valve were identified using the short-axis view (*Figure 1D*) and further assessed using the long-axis view (*Figure 1E*) to confirm anterior mitral leaflet (AML) and posterior mitral leaflet (PML) calcification.

### Data collection and clinical outcome

Baseline clinical data, procedural characteristics, and follow-up data were retrospectively collected from the hospital medical records. Routine clinical follow-ups were performed 1 month, 1 year, and annually after TAVI. The primary endpoint was a composite of all-cause death and heart failure hospitalization and stroke according to Valve Academic Research Consortium (VARC) 3 after TAVI.^17^ Clinical outcomes were collected using records from the institution’s integrated data system, documents provided by the referring physicians, and telephone interviews based on prior consent.

### Statistical analyses

Continuous data were assessed using the Shapiro–Wilk test for the presence or absence of a normal distribution and are presented as the mean (standard deviation) when variables are normally distributed and as the median (interquartile range) when variables are not normally distributed. Categorical data are presented as frequencies or percentages. Continuous variables were compared between groups using the Student *t*-test or Wilcoxon rank-sum test, based on the normality of the data. The Student *t*-test was applied for normally distributed variables, while the Wilcoxon rank-sum test was used for non-normally distributed variables. Categorical data were compared between groups using the *χ*^2^ test. Logistic regression analysis was performed to identify variables associated with true MS. The independent variables included in the multivariate analysis were those considered to be associated with the outcomes based on the findings of previous studies and clinical judgement. The prognostic data were censored on 30 June 2024. Patients lost to follow-up were censored at the date of the last contact/follow-up; patients alive on 30 June 2024 were censored for event-free survival analysis. Event-free survival was defined as post-TAVI until the date of the primary endpoint. Event-free survival was estimated using the Kaplan–Meier method, and survival estimates were compared using the log-rank test. Cox regression analysis, both univariate and multivariate, was performed to assess the prognostic impact of true MS in this population. Specifically, the Society of Thoracic Surgeons score, a comprehensive clinical risk score, was included as a variable in both univariate and multivariate analyses. The proportional hazards assumption for the Cox model was tested and confirmed to be valid. The results are presented as odds ratios (ORs) [95% confidence intervals (CIs)] and hazard ratios (HRs) (95% CIs). Tests were two-sided, with *P* < 0.05 considered statistically significant. In cases where certain values were missing, a complete case analysis was performed. All statistical analyses were performed using JMP Pro 16 software (SAS Institute, Cary, NC, USA).

## Results

### Study population and baseline characteristics

**Figure 2:**
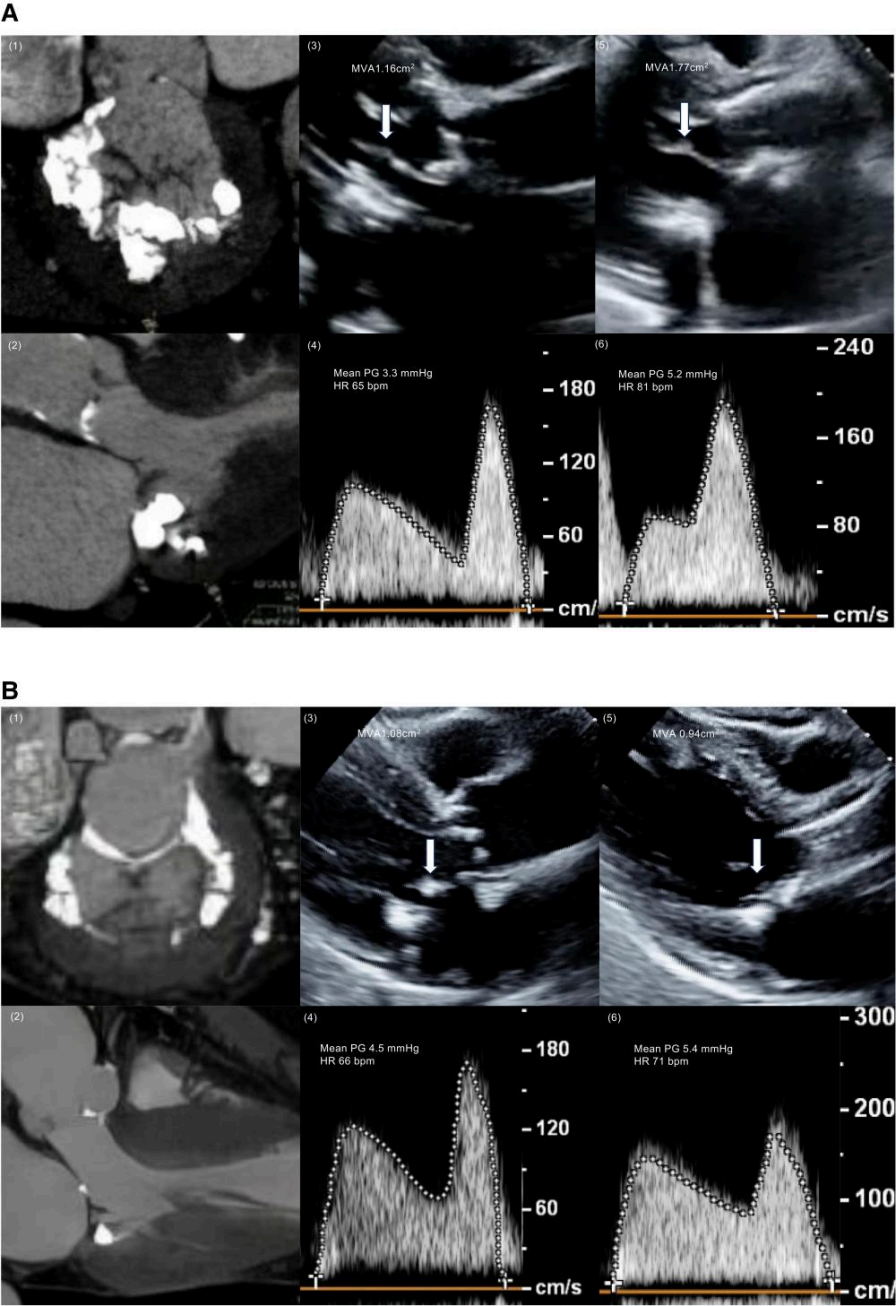
Representative cases of the true and pseudo-MS. (*A*) A typical case of the pseudo-MS group: CT shows severe MAC (1) but no calcification on the AML (2). TTE shows a MVA of 1.16 cm² and a mean pressure gradient (PG) of 3.3 mmHg before TAVI (3, 4). In this case, the AML opening was significantly increased after TAVI (3, 5), leading to an increase in MVA to 1.77 cm² and a slight increase in the mean PG to 5.2 mmHg after TAVI (5, 6). (*B*) A typical case of the true MS group: in addition to severe MAC, AML calcification was also observed (2). TTE showed that AML opening did not change before and after TAVI (3, 5), leading to no MVA increase and a slight increase in the mean PG after TAVI (4, 6).

In the TAVI cohort, degenerative MS was observed in 72 (9.5%) patients. Of these 72 patients, 38 (52.7%) had true MS, while the remaining had pseudo- or mild MS. Baseline clinical characteristics are presented in *Table 1*. No significant differences were observed in the clinical characteristics between the two groups. TTE and CT characteristics are presented in *Table 2*. LV ejection fraction (LVEF), stroke volume (SV), and AS severity did not differ between the two groups. Compared with the mild or pseudo-MS group, the true MS group had a significantly lower MVA and higher TMG [MVA: 1.10 (0.90–1.31) vs. 1.41 (1.20–1.65) cm^2^, *P* < 0.001; TMG: 4.2 (3.6–5.9) vs. 3.3 (2.6–4.1) mmHg, *P* < 0.001]. Moreover, the true MS group had less mild MS and more severe MS, and no difference was observed in moderate MS [mild MS: 5 (13%) vs. 13 (38%), *P* = 0.03; moderate MS: 17 (45%) vs. 20 (59%), *P* = 0.16; severe MS: 16 (42%) vs. 1 (3%), *P* < 0.001]. Patients in the true MS group exhibited significantly higher values of diastolic function parameters, such as E-wave velocity, A-wave velocity, and E/e′ ratio [E-wave velocity: 128 (97–150) vs. 97 (75–116) cm/s, *P* < 0.001; A-wave velocity: 152 (133–170) vs. 128 (118–150) cm/s, *P* < 0.001; E/e′: 31.2 (24.0–40.2) vs. 25.6 (19.0–29.2), *P* = 0.004]. Although CT findings showed no difference in the severity of MAC between the two groups [moderate or severe MAC: 29 (76%) vs. 23 (68%), *P* = 0.41], the true MS group had more PML and AML calcification than the mild or pseudo-MS group [PML calcification: 30 (79%) vs. 19 (56%), *P* = 0.04; AML calcification: 26 (68%) vs. 7 (21%), *P* < 0.001]. Representative cases of true and pseudo-MS are shown in *Figure 2*.

**Table 1:** Comparison of baseline characteristics

	True MS (*n* = 38)	Mild or pseudo-MS (*n* = 34)	*P*-value
Age	84 (82–86)	84 (77–87)	0.44
Sex (male)	7 (18%)	9 (26%)	0.41
BMI, kg/m^2^	22.5 ± 3.5	21.8 ± 3.4	0.35
BSA, m^2^	1.42 ± 0.14	1.44 ± 0.14	0.52
NYHA Class III or IV	12 (32%)	17 (50%)	0.11
Clinical frailty scale	4.0(3.0–5.0)	4.0 (3.0–5.0)	0.87
High surgical risk (STS PROM ≥ 8)	6 (16%)	12 (35%)	0.06
Hypertension	34 (89%)	27 (79%)	0.43
Diabetes	13 (39%)	10 (26%)	0.23
Dyslipidaemia	15 (41%)	8 (24%)	0.12
Chronic kidney disease	20 (57%)	22 (65%)	0.52
Dialysis	2 (5%)	6 (18%)	0.10
Smoking	7 (19%)	11 (32%)	0.21
Chronic lung disease	1 (3%)	4 (12%)	0.13
Atrial fibrillation	7 (18%)	6 (18%)	0.93
Cerebrovascular disorder	5 (15%)	7 (21%)	0.49
ACE inhibitor/ARB	18 (47%)	11 (32%)	0.19
β-Blocker	17 (45%)	12 (35%)	0.41
Ca blocker	19 (50%)	18 (53%)	0.80
Statin	14 (37%)	14 (41%)	0.71
Hob, g/dL	11.0 ± 1.3	11.4 ± 1.7	0.21
Cr, mg/dL	0.94 (0.71–1.14)	0.86 (0.73–1.56)	0.84
eGFR, mL/min/1.73 m^2^	45.7 (35.4–60.1)	47.4 (26.3–66.0)	0.89
LDL, mg/dL	101 (89–119)	100 (85–122)	0.70
HDL, mg/dL	53 (44–65)	59 (48–68)	0.18
HbA1c, %	5.7 (5.6–6.1)	5.7 (5.5–6.4)	0.68
NT-proBNP, pg/mL	1500 (841–3407)	2219 (745–11 433)	0.40
Valve type (balloon expandable)	29 (76%)	27 (79%)	0.75
Valve size			0.24
20 mm	6 (16%)	1 (3%)	0.053
23 mm	20 (53%)	18 (53%)	0.98
26 mm	11 (29%)	14 (41%)	0.28
29 mm	1 (3%)	1 (3%)	0.94
Use of circulatory assist device	1 (3%)	3 (9%)	0.24
Intraoperative complications	1 (3%)	1 (3%)	0.94

Data are presented as the *n* (%), median (interquartile range), or mean (standard deviation, SD).

ACE, angiotensin-converting enzyme; BMI, body mass index; BSA, body surface area; Ca, calcium; Cr, creatinine; eGFR, estimated glomerular filtration rate; Hob, haemoglobin; HDL, high-density lipoprotein; LDL, low-density lipoprotein; NT-proBNP, N-terminal pro-brain natriuretic peptide; NYHA, New York Heart Association; STS PROM, Society of Thoracic Surgeon Predicted Risk of Mortality; TAVI, transcatheter aortic valve implantation.

**Table 2:** Comparison of echocardiographic and CT data

	True MS (*n* = 38)	Mild or pseudo-MS (*n* = 34)	*P*-value
SBP, mmHg	129 (114–154)	131 (121–153)	0.56
DBP, mmHg	61 (54–76)	64 (58–78)	0.16
HR, bpm	70 (62–75)	73 (64–79)	0.45
LVEF, %	68 (62–73)	64 (55–71)	0.06
SV, mL	51 (44–61)	52 (47–59)	0.88
SV index, mL/m^2^	37.0 (31.0–41.0)	37.0 (31.0–42.3)	0.95
LVDd, mm	41 (38–44)	42 (39–51)	0.15
LVDs, mm	26 (23–27)	29 (24–34)	0.03
IVS, mm	11 (9–12)	10 (9–12)	0.33
PWD, mm	11 (9–12)	10 (9–12)	0.73
LAD, mm	43 (39–46)	41 (35–45)	0.17
AVA, cm^2^	0.49 (0.41–0.65)	0.54 (0.42–0.64)	0.52
AVA index, cm^2^/m^2^	0.37 (0.29–0.45)	0.39 (0.30–0.44)	0.50
Peak V, m/s	4.4 (3.8–4.9)	4.4 (3.6–4.9)	0.85
Peak PG, mmHg	77 (56–94)	79 (53–96)	0.85
Mean PG, mmHg	43 (34–55)	42 (30–56)	0.70
MVA, cm^2^	1.10 (0.90–1.31)	1.41 (1.20–1.65)	<0.001
MS severity			<0.001
Mild	5 (13%)	13 (38%)	0.03
Moderate	17 (45%)	20 (59%)	0.16
Severe	16 (42%)	1 (3%)	<0.001
TMG, mmHg	4.2 (3.6–5.9)	3.3 (2.6–4.1)	<0.001
E-wave velocity, cm/s	128 (97–150)	97 (75–116)	0.005
A-wave velocity, cm/s	152 (133–170)	128 (118–150)	0.001
e′-wave velocity, cm/s	3.5 (3.0–4.0)	3.8 (3.0–4.5)	0.61
E/A	0.73 (0.58–1.00)	0.72 (0.60–0.84)	0.45
E/e′	31.2 (24.0–40.2)	25.6 (19.0–29.2)	0.004
LAVI, mL/m^2^	63.6 (49.2–79.7)	53.6 (44.0–72.4)	0.07
TRPG, mmHg	31 (26–39)	34 (24–41)	0.71
Moderate or severe TR	8 (21%)	5 (15%)	0.48
Agatston score of aortic valve	1539 (776–2193)	1761 (1042–2846)	0.21
Moderate or severe MAC, %	29 (76%)	23 (68%)	0.41
PML calcification, %	30 (79%)	19 (56%)	0.04
AML calcification, %	26 (68%)	7 (21%)	<0.001

Data are presented as the *n* (%) or median (interquartile range). Due to the presence of atrial fibrillation patients in whom A-wave could not be measured, A-wave velocity and E/A had missing values in 16 cases (22%).

AML, anterior mitral leaflet; AS, aortic stenosis; AV, aortic valve; AVA, aortic valve area; DBP, diastolic blood pressure; HR, heart rate; IVS, interventricular septum; LAD, left atrial; LAVI, left atrial volume index; LVDd, left ventricular diastolic diameter; LVDs, left ventricular systolic diameter; LVEF, left ventricular ejection fraction; MAC, mitral annular calcification; MVA, mitral valve area; PG, pressure gradient; PML, posterior mitral leaflet; PWD, posterior wall diameter; RVSP, right ventricular systolic pressure; SBP, systolic blood pressure; SV, stroke volume.

### Changes in TTE findings after TAVI

**Figure 3:**
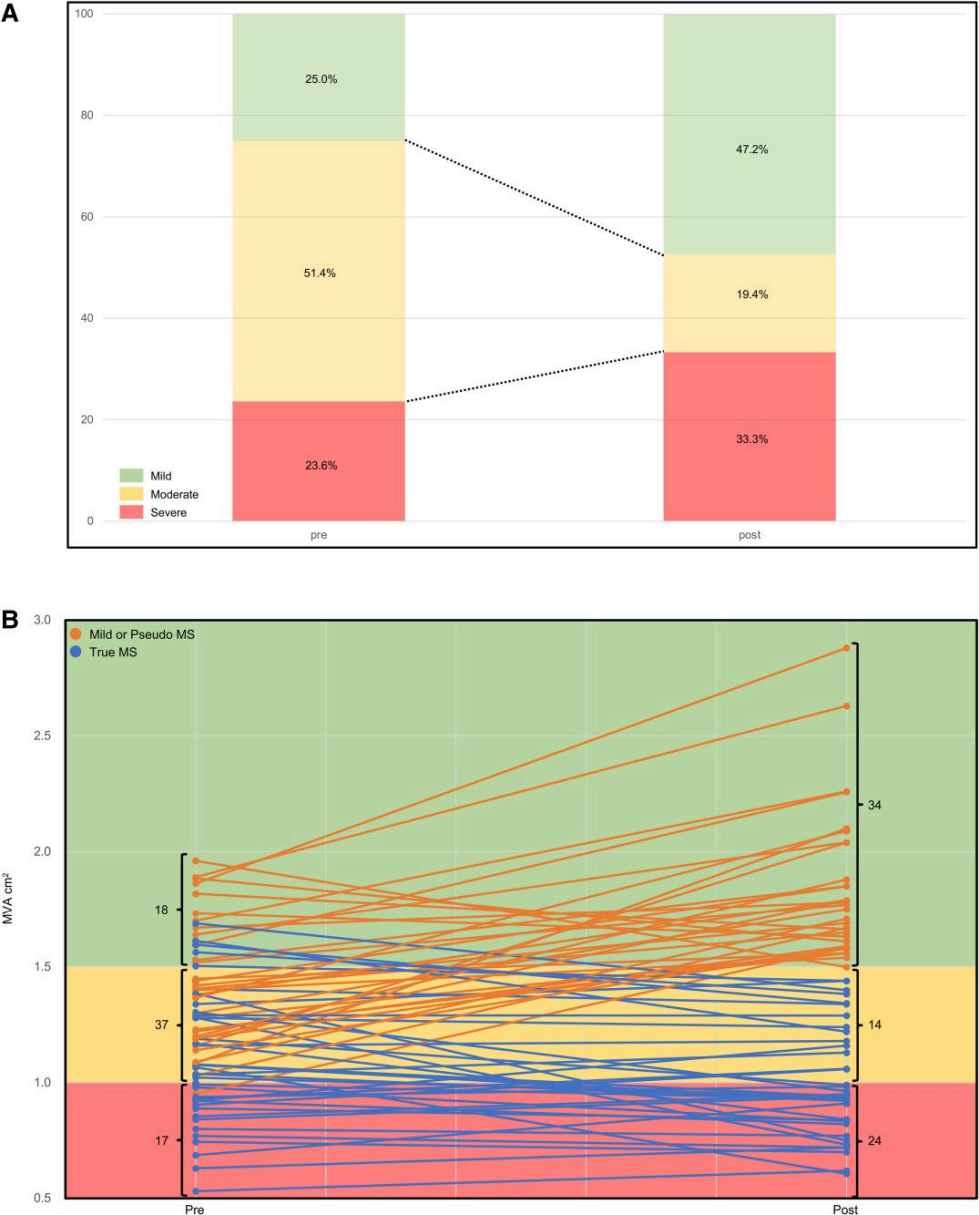
Changes in MS pre- and post-TAVI. (*A*) Change in percentage of each severity. (*B*) Change in the number of cases of each severity and the change in MVA in each case.

*Table 3* presents the changes in the main TTE findings in both groups before and after TAVI. Compared with the mild or pseudo-MS group, the true MS group had smaller SV after TAVI and a smaller change from pre-TAVI to post-TAVI discharge [SV: 49.5 (43.0–58.0) vs. 61.0 (52.0–72.3) mL, *P* < 0.001; ΔSV: −3.5 (−7.4–1.0) vs. 11.0 (−2.5–19.0) mL, *P* < 0.001]. The true MS group also had a smaller effective orifice area index (EOAi) and ΔEOAi [EOAi: 0.81 (0.71–0.96) vs. 1.10 (0.88–1.37) cm², *P* < 0.001; ΔEOAi: 0.56 (0.47–0.68) vs. 0.83 (0.64–1.13) mL, *P* < 0.001]. The mild or pseudo-MS group had higher TMG, tricuspid regurgitation pressure gradient (TRPG), and ΔTRPG [TMG: 4.8 (3.3–6.4) vs. 3.8 (3.0–5.0) mmHg, *P* = 0.03; TRPG: 32.7 (28.2–43.5) vs. 28.9 (26.0–33.4), *P* = 0.03; ΔTRPG: 2.7 (−3.4–6.7) vs. −1.7 (−10.3–2.2) mmHg, *P* = 0.02]. In some patients undergoing TAVI, the severity of MS changed pre- and post-TAVI. *Figure 3A* shows the change in percentage of each severity, and *Figure 3B* shows the change in the number of cases of each severity and the change in MVA in each case.

**Table 3:** Echocardiographic changes after TAVI

	True MS (*n* = 38)	Mild or pseudo-MS (*n* = 34)	*P*-value
SV, mL	49.5 (43.0–58.0)	61.0 (52.0**–**72.3)	<0.001
ΔSV, mL	−3.5 (−7.4**–**1.0)	11.0 (−2.5**–**19.0)	<0.001
EOA, cm^2^	1.14 (1.01**–**1.28)	1.61 (1.31**–**1.91)	<0.001
ΔEOA, cm^2^	0.81 (0.60**–**0.94)	1.23 (0.90**–**1.54)	<0.001
EOAi, cm^2^/m^2^	0.81 (0.71**–**0.96)	1.10 (0.88**–**1.37)	<0.001
ΔEOAi, cm^2^/m^2^	0.56 (0.47**–**0.68)	0.83 (0.64**–**1.13)	<0.001
Max velocity, m/s	2.2 (1.9**–**2.5)	2.0 (1.8**–**2.4)	0.31
Peak PG, mmHg	19.6 (14.2**–**24.0)	16.0 (12.9**–**22.4)	0.31
Mean PG, mmHg	11.0 (8.0**–**13.2)	8.2 (7.0**–**11.3)	0.13
MVA, cm^2^	0.94 (0.81**–**1.19)	1.75 (1.56**–**2.04)	<0.001
ΔMVA, cm^2^	−0.06 (−0.21**–**0.09)	0.40 (0.25**–**0.65)	<0.001
ΔMVA ≥0.1 cm^2^	4 (11%)	30 (88%)	<0.001
TMG, mmHg	4.8 (3.3**–**6.4)	3.8 (3.0**–**5.0)	0.03
ΔTMG, mmHg	0.5 (−0.3**–**1.4)	0.4 (−0.4**–**1.1)	0.78
TRPG, mmHg	32.7 (28.2**–**43.5)	28.9 (26.0**–**33.4)	0.03
ΔTRPG, mmHg	2.7 (−3.4**–**6.7)	−1.7 (−10.3**–**2.2)	0.02
Moderate or severe TR	8 (22%)	5 (15%)	0.42

Data are presented as the *n* (%) or median (interquartile range). The symbol ‘Δ’ stands for ‘change’.

EOA, effective orifice area; EOAi, effective orifice area index; MVA, mitral valve area; PG, pressure gradient; SV, stroke volume; TMG, trans-mitral gradient; TR, tricuspid regurgitation; TRPG, tricuspid regurgitation pressure gradient.

### Factors associated with true MS

Univariate and multivariate logistic regression analyses were performed to identify independent factors statistically associated with true MS before TAVI (*Table 4*). The multivariate analysis identified a statistically significant association between true MS and a smaller MVA (adjusted OR, 1.29, 95% CI = 1.02–1.63). Additionally, AML calcification was identified as an independent factor associated with true MS (adjusted OR, 9.23; 95% CI = 2.84–29.9, *P* < 0.001).

**Table 4:** Variables associated with true MS

	Univariate		Multivariate: Model 1		Multivariate: Model 2	
	OR (95% CI)	*P*-value	Adjusted OR (95% CI)	*P*-value	Adjusted OR (95% CI)	*P*-value
Echocardiographic variables						
SV index per 1 mL/m^2^	1.00 (0.95–1.05)	0.88				
MVA per 0.1 cm^2a^	1.45 (1.19–1.76)	<0.001	1.29 (1.02–1.63)	0.02		
TMG per 1 mmHg	1.81 (1.20–2.72)	<0.001	1.40 (0.86–2.27)	0.14		
DVI (MV VTI/LVOT VTI) ≧ 2.5	3.47 (0.94–12.8)	0.06				
E-wave velocity, cm/s	1.02 (1.01–1.04)	0.009	1.00 (0.98–1.03)	0.54		
E/e′	1.09 (1.02–1.15)	0.007	1.01 (0.93–1.10)	0.78		
LAVI, mL/m^2^	1.02 (0.99–1.05)	0.06				
TRPG per 1 mmHg	1.00 (0.96–1.05)	0.85				
CT variables						
Moderate or severe MAC	1.54 (0.55–4.35)	0.41			0.48 (0.12–1.81)	0.27
PML calcification	2.96 (1.05–8.32)	0.04			2.91 (0.83–10.2)	0.10
AML calcification	8.36 (2.85–24.5)	<0.001			9.23 (2.84–29.9)	<0.001

Model 1 was adjusted for the MVA, TMG, E-wave velocity, and E/e′. Model 2 was adjusted for moderate and severe MAC, PML, and AML calcification.

AML, anterior mitral leaflet; CI, confidence interval; CT, computed tomography; DVI, Doppler velocity index; LAVI, left atrial volume index; LVOT, left ventricular outflow tract; MAC, mitral annular calcification; MV, mitral valve; MVA, mitral valve area; OR, odds ratio; PML, posterior mitral leaflet; SV, stroke volume; TMG, trans-mitral gradient; TRPG, tricuspid regurgitation pressure gradient; VTI, velocity–time integral.

^a^A decrease per unit.

### Clinical outcomes

**Figure 4:**
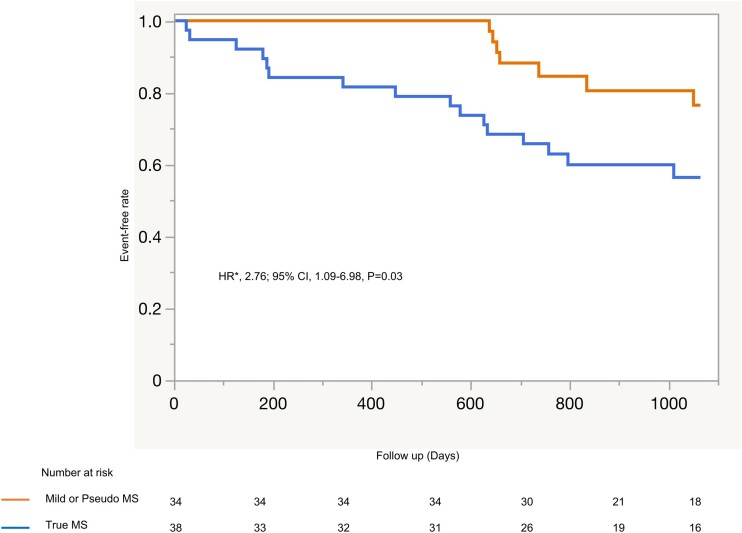
Event-free survival curve for the composite endpoint of all-cause death, heart failure hospitalization, and stroke. *Adjusted for Society of Thoracic Surgeon Predicted Risk of Mortality. CI, confidence interval; HR, hazard ratio; MS, mitral stenosis.

During the 3-year follow-up after TAVI, 24 events were observed in the entire cohort: 12 (16.7%) patients died, 9 (12.5%) were hospitalized for heart failure, and 3 (4.2%) had strokes. The true MS group had a significantly lower event-free survival rate at 3 years compared with the mild or pseudo-MS group (56.4 ± 8% vs. 76.5 ± 8%, log-rank *P* = 0.04). In a multivariate Cox regression analysis, true MS was identified as an independent poor prognostic factor in this population (HR 2.76, 95% CI 1.09–6.98, *P* = 0.03; *Figure 4*). The detailed results of the Cox regression analysis are presented in Supplementary data online, *Table S1*. We performed an additional analysis including a reference group of patients undergoing TAVI without MS. The Kaplan–Meier curve is presented in Supplementary data online, *Figure S1*. In a multivariate Cox regression analysis, true MS was identified as an independent poor prognostic factor (HR 1.94, 95% CI 1.15–3.27, *P* = 0.01; Supplementary data online, *Table S2*). However, mild or pseudo-MS was not associated with poor prognosis.

## Discussion

Concomitant degenerative MS in patients undergoing TAVI presents a unique clinical challenge. The interaction between AS and MS complicates the accurate assessment of the severity of each condition, which, in turn, affects treatment strategies. The major findings of this study, which examined the recent focus on degenerative MS in the TAVI cohort, are as follows: (i) degenerative MS was present in 9% of the entire TAVI cohort, indicating that it is not uncommon; (ii) among patients with concomitant degenerative MS undergoing TAVI, approximately half had true MS; (iii) this study revealed the rate and extent of changes in MS severity following TAVI, attributable to the haemodynamic improvements induced by the procedure; (iv) true MS was associated with a poor prognosis; and (v) AML calcification on CT was useful for predicting true MS.

### Predictors of true MS

Our analysis identified AML calcification as a significant predictor of true MS. This finding underscores the role of CT imaging in the comprehensive evaluation of mitral valve morphology before TAVI. The detailed assessment of mitral valve calcification using multi-detector CT allowed for the differentiation between true and pseudo-MS. Echocardiographic assessment of mitral valve characteristics in patients with degenerative MS is difficult because of calcification artefacts. Therefore, to identify true MS in patients with degenerative MS undergoing TAVI, we investigated not only echocardiographic findings but also CT findings before the TAVI procedure. Specifically, AML calcification was significantly more prevalent in the true MS group than in the mild or pseudo-MS group. There was a considerable difference in that patients with true MS exhibited significantly higher TMG and poorer clinical outcomes. Previous studies have reported a correlation between AML calcification and the MVA in patients with degenerative MS.^18^ Degenerative MS patients with AML calcification also reportedly had increased TMG and decreased MVA after TAVI.^19^ In our TAVI cohort, we observed improved AML mobility after TAVI in typical cases of mild or pseudo-MS; however, this phenomenon was not observed in typical patients with true MS (*Figure 2*). In addition, the ΔSV was associated with changes in the MVA before and after TAVI. These observations may be explained by the haemodynamic changes caused by TAVI and the fact that the AML has a longer valve length than the PML and is composed of a single scallop, unlike the PML, which comprises three scallops.

### Clinical outcomes

True MS was associated with a significantly lower event-free survival rate at 3 years post-TAVI compared with mild or pseudo-MS. This finding aligns with that of previous studies that have reported poor prognosis in patients with severe MS undergoing TAVI.^4,8,20^ The higher TMG and smaller EOAi observed in the true MS group post-TAVI likely contributed to the adverse outcomes. The persistence of significant MS despite successful TAVI emphasizes the need for careful preoperative assessment and post-procedural monitoring.

### Clinical implications

In cases of combined MS and AS, it is often difficult to determine which condition has a greater impact on the patient’s clinical presentation. When there is a discrepancy between the severity of symptoms and the apparent degree of stenosis, stress echocardiography is recommended for assessing MS.^6^ There is evidence that exercise-induced pulmonary hypertension, as evaluated by stress echocardiography, serves as a prognostic factor in MS patients,^21^ highlighting its potential value in such cases. However, the role of stress echocardiography in patients with both MS and AS remains unclear, and future studies and data accumulation are necessary. It is also important to recognize the limitations of stress echocardiography, particularly in patients with severe comorbidities or in cases where the presence of multiple valve diseases complicates interpretation. The identification of true MS has significant implications in clinical practice. In our study, some patients with degenerative MS who underwent TAVI showed a change in the severity of their MS after TAVI, masking the preoperative severity. Notably, this change was more pronounced in moderate MS, which is considered clinically significant MS.^6^ Given the poor prognosis associated with true MS, it is essential to incorporate detailed imaging analyses, such as CT, into the preoperative assessment of patients with AS undergoing TAVI. By identifying patients with true MS during pre-TAVI planning using echocardiography and CT, physicians can better stratify risks and tailor management strategies to improve outcomes. For example, the presence of AML calcification could prompt the consideration of more aggressive or alternative interventions to address both AS and MS.

### Study limitations

This study had some limitations. First, this study used MVA derived from the continuity equation. Given the inherent measurement error in this method, low cut-off values may fall within the range of observational error. Therefore, we conducted a sensitivity analysis using a ΔMVA of 0.2 cm² as the definition of true MS without considering the severity of MS (see Supplementary data online, *Figure S2*). The results confirmed that true MS remained an independent poor prognostic factor at this cut-off (HR, 2.91; 95% CI = 1.07–7.87, *P* = 0.04). The primary result of this study, that AML calcification is an independent factor associated with true MS, was also upheld (adjusted OR 10.3, 95% CI =2.99–35.3, *P* < 0.001). These findings support the importance of our conclusions. Second, it was a retrospective observational study, which may have introduced a selection bias. Third, this study was conducted at a single centre and included a relatively small sample size, which may limit the generalizability of our findings. Fourth, the follow-up period, although relatively long, may not have captured all long-term outcomes. Future prospective multicentre studies are needed to validate our findings and explore the long-term impact of true MS in patients who have undergone TAVI.

## Conclusions

Approximately half of the patients with concomitant degenerative MS undergoing TAVI had true MS, which was associated with a poor prognosis. CT analysis of AML calcification was useful for predicting true MS.

## Supplementary Material

qyae109_Supplementary_Data

## Data Availability

The data underlying this article were provided by St. Marianna University Hospital. Data will be shared on request to the corresponding author with permission of St. Marianna University Hospital.

## Lead author biography

Mituski Yamaga is a cardiologist currently enrolled in the Graduate School of Medicine at St. Marianna University School of Medicine in Kawasaki, Japan. His research focuses on cardiovascular imaging, including echocardiography and CT, as well as structural heart disease.
