# Supplementary material for: Predictors and clinical outcomes of true mitral stenosis in patients undergoing transcatheter aortic valve implantation
Source: Eur Heart J Imaging Methods Pract. 2024 Oct 23;2(3):qyae109. doi: 10.1093/ehjimp/qyae109 (PMC11551227; doi:10.1093/ehjimp/qyae109)
Supplement: qyae109_Supplementary_Data [file qyae109_supplementary_data.zip › Supplementary Legends.docx]

**Supplementary Figure 1. Event-free survival curve for the true MS, mild or pseudo-MS and non-MS**

The endpoint is a composite of all-cause death, heart failure hospitalization and stroke. CI, confidence interval; HR, hazard ratio; MS, mitral stenosis

**Supplementary Figure 2. Event-free survival curve for true MS and mild or pseudo-MS using a cut-off value of ΔMVA = 0.2 cm².**

*Adjusted for Thoracic Surgeon-Predicted Risk of Mortality. The endpoint is a composite of all-cause death, heart failure hospitalization and stroke. When the analysis was also performed using a cut-off value of ΔMVA = 0.2 cm²**,** AML calcification was identified as an independent factor associated with true MS (adjusted OR 10.3, 95% CI=2.99‒35.3, P<0.001). AML, anterior mitral leaflet; CI, confidence interval; HR, hazard ratio; MS, mitral stenosis; MVA, mitral valve area.

**Supplementary Table 1. Variables associated with primary endpoint in degenerative MS cohort**

| Variable | Univariate | | Multivariate | |
| --- | --- | --- | --- | --- |
|  | HR (95% CI) | P value | HR (95% CI) | P value |
| True MS | 2.51 (1.03–6.11) | 0.04 | 2.76 (1.10–6.98) | 0.03 |
| High surgical risk (STS PROM ≥8) | 1.09(0.43–2.77) | 0.85 | 1.49(0.56–3.93) | 0.42 |

CI, confidence interval; HR, hazard ratio; MS, mitral stenosis; STS PROM, Society of Thoracic Surgeons Predicted Risk of Mortality.

**Supplementary Table 2. Variables Associated with primary endpoint in including non-MS cohort**

| Variable | Univariate | | Multivariate | |
| --- | --- | --- | --- | --- |
|  | HR (95% CI) | P value | HR (95% CI) | P value |
| True MS | 1.87 (1.11–3.15) | 0.02 | 1.94 (1.15–3.27) | 0.01 |
| Mild or Pseudo MS | 0.87 (0.42–1.78) | 0.70 | 0.76 (0.37–1.57) | 0.46 |
| High surgical risk (STS PROM ≥8) | 1.89(1.33–2.68) | <0.001 | 1.95(1.37–2.78) | <0.001 |

CI, confidence interval; HR, hazard ratio; MS, mitral stenosis; STS PROM, Society of Thoracic Surgeons Predicted Risk of Mortality.
