# Supplementary figures and images for: Predictors and clinical outcomes of true mitral stenosis in patients undergoing transcatheter aortic valve implantation
Source: Eur Heart J Imaging Methods Pract. 2024 Oct 23;2(3):qyae109. doi: 10.1093/ehjimp/qyae109 (PMC11551227; doi:10.1093/ehjimp/qyae109)

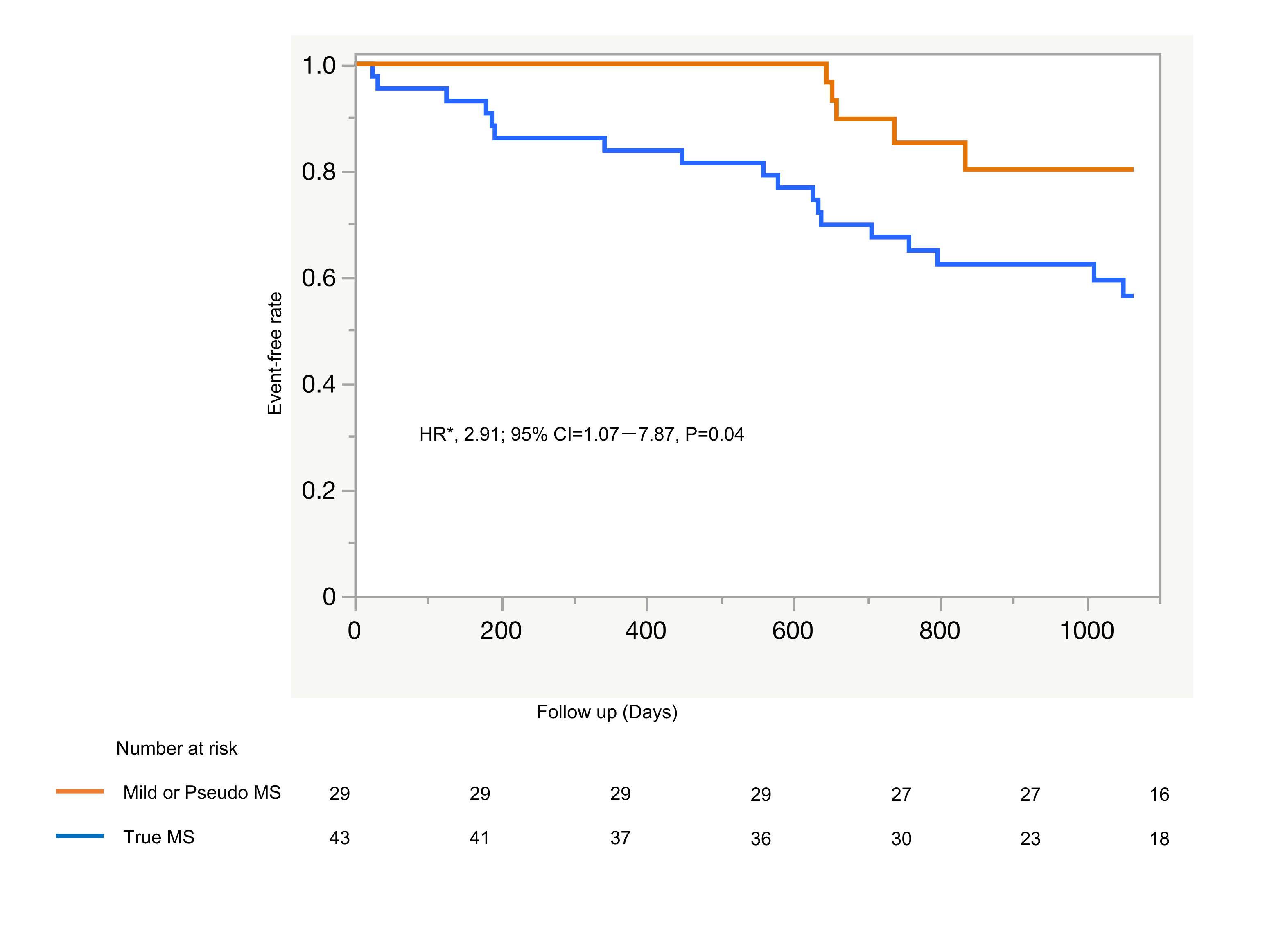

Supplement: qyae109_Supplementary_Data [file qyae109_supplementary_data.zip › Supplemental Figure 2.tif]

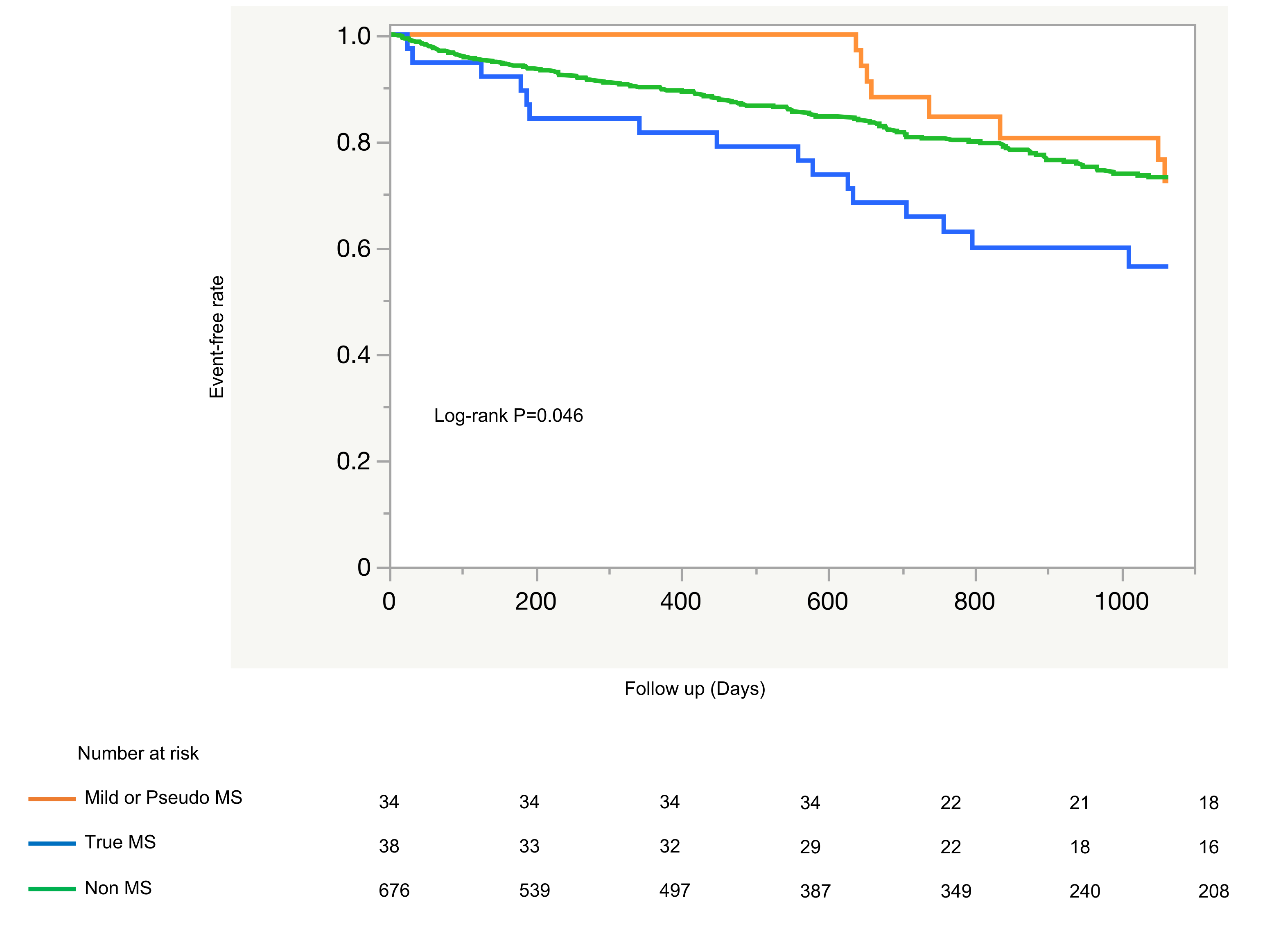

Supplement: qyae109_Supplementary_Data [file qyae109_supplementary_data.zip › Supplemental Figure 1.tif]
